# A New Catalog of Microbiological Tools for Women's Infectious Disease Research

**DOI:** 10.1128/genomeA.00890-16

**Published:** 2016-09-29

**Authors:** Amanda L. Lewis, Warren G. Lewis

**Affiliations:** a Department of Molecular Microbiology, Washington University School of Medicine, St. Louis, Missouri, USA; b Department of Obstetrics and Gynecology, Washington University School of Medicine, St. Louis, Missouri, USA; c Department of Internal Medicine, Washington University School of Medicine, St. Louis, Missouri, USA; d Division of Infectious Disease, Washington University School of Medicine, St. Louis, Missouri, USA; e Center for Women’s Infectious Disease Research, Washington University School of Medicine, St. Louis, Missouri, USA

## Abstract

Genitourinary infections pose serious health risks. But, little is known about how genitourinary bacteria attach, maintain colonization, compete for resources, and cause pathology. In this issue, we introduce a new set of 62 genitourinary reference strains of bacteria and their genomes to spur experimental research on infectious diseases that impact women.

## COMMENTARY

Bacteria that inhabit niches in the female genitourinary system play vital roles in health and disease throughout the female life span. Species of *Lactobacillus* are often abundant components of the vaginal microbiota in reproductive-age women and are widely regarded as “healthy” vaginal bacteria. In contrast, it is estimated that one-third of women in the United States have bacterial vaginosis (BV) (1), an imbalance of the vaginal microbiota marked by few lactobacilli and overgrowth of potentially pathogenic anaerobic microorganisms, including Gram-negative bacteria, *Firmicutes*, and *Actinobacteria*. BV is associated with higher risks of health complications, such as the acquisition of sexually transmitted infections (including HIV) (2–4), urinary tract infections (5, 6), pelvic inflammatory disease, infertility, infectious complications of gynecological surgeries, and pregnancy complications, such as preterm birth (7, 8), placental infection (9, 10), and amniotic fluid infection (11). In fact, several genera of BV-associated bacteria have been isolated from sites of infection in the upper reproductive tract, including species of *Gardnerella, Prevotella, Fusobacterium, Megasphaera*, and others (12–14). Despite the clear obstetric and gynecologic health risks associated with BV, we still know surprisingly little about the underlying causes of the condition. Available antibiotic therapies for BV appear to be initially effective in many cases; however, recurrence rates are high (4, 5). Still, the molecular ecological mechanisms that help explain the condition’s frequent recurrence (15) and the specific culprits within the BV community that engender disease have been challenging to pursue.

Several interdependent factors have contributed to the ill-defined roles of urogenital bacteria in infectious diseases of the female urinary and reproductive tracts, including BV. Perhaps the most significant among these factors is that the genital tract has been a neglected area of study relative to other host-microbial interfaces, such as the gastrointestinal and respiratory tracts. It is therefore not altogether surprising that the experimental tools for investigating host-microbe interactions in this niche are underdeveloped. The isolation and culture of many of the fastidious anaerobes of the female genital tract can be challenging. Thus, a major barrier to progress in the field is the limited availability of well-characterized vaginal bacterial isolates. Research in this area is further constrained by insufficient coverage of taxonomic diversity among vaginal bacterial genomes in public databases, which places major limitations on metagenomic, metatranscriptomic, and metaproteomic approaches applied to human vaginal specimens. All in all, the availability of new strains of taxonomically diverse vaginal bacteria and their genomes should help overcome major barriers in this area of study.

In this issue, we announce the completion of high-quality draft genomes for 61 new strains of urogenital bacteria, including 9 strains representing five genera of Gram-negative bacteria, 14 strains representing 11 genera in the phylum *Firmicutes*, and 26 strains representing seven genera of the phylum *Actinobacteria* (16–20). Both the genome sequences and the reference strains of bacteria have been made available to the research community. The strains have been deposited with BEI Resources.

*Firmicutes* are among the most common bacteria in the vagina and include examples of both pathogenic and commensal microorganisms. Species of *Lactobacillales* (including vaginal lactobacilli) are well represented in culture collections and genome sequencing efforts. However, there are many fewer available vaginal isolates and genomes of *Firmicutes* that are associated with BV. Here, we put forward isolates and genomes of BV-associated anaerobes in the genera *Anaerococcus, Finegoldia, Peptoniphilus*, and *Peptostreptococcus*, as well as two strains of the diderm (2-membrane) *Negativicutes*:*Megasphaera* and *Veillonella*. We have also isolated and sequenced vaginal strains of commensal and potentially pathogenic *Staphylococcus, Streptococcus, Clostridium,* and *Enterococcus* species*.* Finally, we also present a new vaginal isolate of *Bacillus coagulans*, a bacterium that has been used as an additive in some vaginal probiotic suppositories.

*Actinobacteria* is a phylum known for its diversity and abundance in soil and for the ability of certain members to synthesize antibiotic compounds and other useful molecules. Within the human body, *Actinobacteria* also come in many varieties. However, we still know relatively little about why some *Actinobacteria* form symbiotic relationships with the human host, while others form relationships better described as commensal, opportunist, or pathogen. Here, we provide 27 new strains and genomes of vaginal *Bifidobacterium, Corynebacterium, Propionibacterium*, *Actinomyces, Alloscardovia, Atopobium,* and *Gardnerella* spp. for further investigation. Recent studies have demonstrated that the *Gardnerella vaginalis* was sufficient to elicit several features of BV in a murine model, including evidence of mucus degradation (21), an epithelial exfoliation response, and the formation of clue-like cells with attached *G. vaginalis* (22). Here, we report the availability of 19 individual isolates of *G. vaginalis*, including strains from women who did not have BV (21), and a strain that was isolated from a clean-catch urine specimen.

In addition to *Firmicutes* and *Actinobacteria*, Gram-negative bacteria are also found to overgrow in the vagina during bacterial vaginosis. However, there are few fully sequenced vaginal isolates of Gram-negative bacteria. In particular, we present several draft genome sequences and strains of the *Bacteroidetes* and *Fusobacteriales*, both of which are common isolates from sites of infection in the upper reproductive tract during pregnancy.

The urinary tract is one of the most common sites of infection in humans (~7 million infections worldwide each year), and the vagina is thought to be a reservoir for uropathogenic bacteria. *Escherichia coli* is the most common cause of urinary tract infections (UTIs) in humans (23), and its cellular and molecular interactions with the mammalian host have been extensively studied. Nevertheless, pathogens other than *E. coli* cause UTIs, and these infections number in the hundreds of thousands each year. Many of these “alternate” uropathogens are poorly understood (24). Here, we present 12 isolates representing 5 genera of bacteria isolated from clean-catch urine samples from pregnant women. For example, *Streptococcus agalactiae* (aka group B *Streptococcus* [GBS]) is a vaginal commensal that sometimes acts as a pathogen, particularly in pregnant women (25), infants (26, 27), and the elderly (28). While best known for its designation as a leading cause of sepsis and meningitis in newborns, GBS also causes UTIs, particularly in pregnant women and the elderly. Interestingly, GBS can also enhance the pathogenesis of *E. coli* UTIs in mouse models (29, 30). Here, we provide seven new strains of GBS isolated from the urinary tracts of pregnant women along with their draft genome sequences. We also present draft genome sequences of several vaginal isolates of Gram-negative bacteria known for their ability to cause UTIs (e.g., *Proteus, Klebsiella*, and *Citrobacter* species).

With these strains in hand, the corresponding genomes offer a rich source of hypotheses and the means to test the roles of bacteria in experimental models of the female urogenital microbiota in health and disease.
